# Analysis of haploinsufficiency in human neural progenitor cells: insights into early molecular effects of autism-related genes

**DOI:** 10.1038/s41419-026-09085-2

**Published:** 2026-07-08

**Authors:** Roni Sarel-Gallily, Assa Sherman, Daniel Pollak, Nissim Benvenisty

**Affiliations:** The Azrieli Center for Stem Cells and Genetic Research, Department of Genetics, Institute of Life Sciences, The Hebrew University, Jerusalem, Israel

**Keywords:** Embryonic stem cells, Pluripotent stem cells

## Abstract

Haploinsufficiency describes a phenomenon where one functional allele of a gene in a diploid cell or organism is insufficient for a normal phenotype. There are several neurodevelopmental disorders (NDD) affected by the haploinsufficiency phenomenon, and many of them are related to autism spectrum disorder (ASD). Here, we aim to identify genes involved in the early stages of neural differentiation when one of the two alleles is lost. We thus differentiated a genome-wide heterozygous loss-of-function CRISPR library into neural progenitor cells (NPCs) and defined about 250 genes essential for neural differentiation in a haploinsufficient manner. We were able to identify NDD-related dosage-sensitive pathways and pinpoint specific molecular processes affected by ASD-related genes. By comparing the molecular phenotypes of homozygote and heterozygote mutations, we could illuminate overlapping and distinct transcriptional pathways affected in the two mutant models, along with partial chemical rescue of some of these phenotypes. Our work provides a comprehensive framework for exploring dosage-sensitive regulation in early neural development and offers new insights into the embryonic molecular basis of ASD and other NDDs driven by gene dosage imbalance.

## Introduction

Human embryonic stem cells (hESCs) are pluripotent cells derived from the inner cell mass of the pre-implantation blastocyst and possess two valuable attributes: the unlimited self-renewal ability in culture and the potential to differentiate into all somatic cell lineages [1]. These unique capabilities make hESCs a powerful tool for studying human development [2], disease modelling [3] and cell therapy [4]. Directed differentiation protocols allow hESCs to recapitulate early embryonic events, enabling analysis of the genetic and molecular mechanisms shaping germ layer differentiation [5].

Haploinsufficiency occurs when a loss-of-function mutation in one out of two alleles causes a phenotype. Haploinsufficiency is especially relevant in the context of neurodevelopmental disorders (NDDs) [6–9], including autism spectrum disorders (ASD) [10, 11], intellectual disabilities [12], and others. Due to the complexity of getting only heterozygous mutations in candidate genes, studies attempting to explore haploinsufficiency have taken several approaches: using animal models [13, 14], deriving induced pluripotent stem cells (iPSCs) from patients [15, 16], focusing on an individual gene or a small number of genes and performing vast CRISPR editing along with colony selection [17, 18] or planning allele-specific sgRNAs [19]. These approaches, while beneficial on several different levels, are not suitable for genome-wide screens. Therefore, the wider effect of gene dosage imbalance on early neural development is still largely unknown.

Genome-wide loss-of-function CRISPR screens have become more common in research as a tool for assessing genes essential for growth, survival and differentiation. We have previously isolated haploid hESCs [20], providing an advantage in establishing a genome-wide loss-of-function CRISPR library in those cells [21] and identified genes essential for hESCs' growth and survival. The library was differentiated into the three embryonic germ layers [22], mapping the genes essential for different differentiation trajectories.

We established a genome-wide CRISPR-Cas9 heterozygous loss-of-function library in hESCs [23], enabling identification of essential haploinsufficient genes in the human pluripotent cell state. These genes were discovered to be located across all chromosomes (although relatively depleted from X chromosome), and were enriched in extracellular matrix and plasma membrane, leading us to further focus on signalling pathway that operate in a dosage-sensitive manner. We revealed that both WNT and TGF-beta pathways were dosage regulated and explored two disease-related haploinsufficiency genes and their transcriptomic effects when mutated, thus identifying dosage-sensitive and dosage-independent signalling pathways in hESCs.

In this study, we utilize this unique library to investigate haploinsufficiency in the context of early neural differentiation. By differentiating the haploinsufficient hESCs library into neural progenitor cells (NPCs), we screened for genes essential for the survival or neuroectodermal differentiation in a haploinsufficient manner.

Our analyses reveal several signalling pathways enriched with NDD-related haploinsufficiency genes. We further focused on ASD-related genes in NPCs and characterize the distinct molecular consequences of haploinsufficiency mutations in them. Together, our findings establish a comprehensive image of gene dosage sensitivity in early neural differentiation and provide mechanistic insight into how heterozygous loss-of-function contributes to human neurodevelopmental diseases.

## Materials and Methods

### Cell lines and cell culture

The following cell lines were used in this study: h-pES10 and h-pES12 cell lines are two haploid hESCs lines previously isolated in our lab [20], h-pES10-GFP: h-pES10 cells expressing GFP fluorophore and neomycin resistance; h-pES12-dsRed: h-pES12 cells expressing dsRed fluorophore and hygromycin resistance; haploinsufficiency diploid mutation library: diploid mutant library previously established in our lab [23] by fusion of wild-type haploid h-pES12-dsRed and haploid mutant library in h-pES10 haploid cell line [21] (based on sgRNA library from Wang et al. [24]). HEK293T cells: human embryonic kidney cells containing the SV40 T-antigen obtained from R. Weinberg (Whitehead Institute).

All hESC lines were cultured at 37 °C and 5%CO_2_ and were plated on Matrigel coated 6-well plates (Corning) in mTeSR1 medium (STEMCELL Technologies). Cells were passaged by trypsinization using trypsin-EDTA (Biological Industries) and plated in the presence of 10 μM ROCK inhibitor Y-27632 (Gentaur) for 1 day after splitting.

Cell lines undergoing infection and CRISPR editing were grown on feeder layer of growth-arrested mouse embryonic fibroblasts (MEFs), containing drug resistance for 4 different drugs: puromycin, hygromycin, neomycin and 6-thioguanine (DR4, Jackson laboratories). The cells were fed with standard hESC growth medium, composed of knockout Dulbecco’s modified Eagle’s medium (DMEM, Thermo Fisher Scientific) supplemented with 15% knockout serum replacement (KSR, Thermo Fisher Scientific), 2 mM L-glutamine (Biological Industries), 0.1 mM nonessential amino acids (Biological Industries), 50 units/ml penicillin, 50 μg/mL streptomycin (Biological Industries), 0.1 mM β-mercaptoethanol (Sigma) and 8 ng/mL basic fibroblast growth factor (bFGF, PeproTech).

DR4 MEF cells were cultured in DMEM with 10% fetal bovine serum (Thermo Fisher Scientific), 2mM L-glutamine, 50 units/ml penicillin and 50 μg/mL streptomycin.

### NPC differentiation

The haploinsufficiency library and mutated clones were differentiated into neural progenitor cells as described previously [22] with minor modifications. Briefly, cells were plated on Matrigel-coated 6-well plates at a density of 1.75 million cells per well and supplemented with mTeSR1 medium. The next day, the medium was replaced by embryonic body (EB) medium (identical to hESCs growth medium, but without bFGF), supplemented with 10 μM of SB431542 (Biogems) and 2.5 μM of LDN193189 (Biogems). EB medium was replaced daily for 4 days. On day 5, EB medium was replaced by a mixture of 3:1 EB medium and N2 medium Dulbecco’s Modified Eagle’s Medium/Nutrient Mixture F-12 Ham (Sigma), 1 x N-2 supplement (Thermo Fisher, cat. no. 17502001), 50 units/ml penicillin, 50 μg/ml streptomycin, 2 mM L-glutamine, 0.1 M ascorbic acid (Sigma) and 0.16% D-Glucose) supplemented with of 10 μM of SB431542 and 2.5 μM of LDN193189. On day 6, medium was changed to a 1:1 ratio mix of EB medium and N-2 medium in the presence of 10 μM of SB431542 and 2.5 μM of LDN193189. On day 7, the medium was changed to a 1:3 mix of EB medium and N-2 medium in the presence of 10 μM of SB431542 and 2.5 μM of LDN193189. On day 8, the medium was changed to N-2 medium in the presence of 10 μM of SB431542 and 2.5 μM of LDN193189. The cells were harvested the following day (day 9) for downstream analyses.

### DNA extraction and sequencing

Genomic DNA was extracted using the Blood & Cell Culture DNA Midi Kit (QIAGEN) according to the manufacturer’s protocol. The sgRNA integration region was amplified using the following primers: Foreward- TCGTCGGCAGCGTCAGATGTGTATAAGAGACAGNNN NNNNNNNGGCTTTATATATCTTGTGGAAAGGACG, reverse- GTCTCGTGGGCTCGGAGATGTGTATAAGAGACAGACG GACTAGCCTTATTTTAACTTGC. Nextera DNA libraries were prepared as described previously [21], and sequencing was performed on the Illumina NextSeq 500 platform.

### RNA extraction, sequencing and analysis

RNA was extracted from confirmed heterozygous, homozygous, and wild-type clones using the NucleoSpin RNA Plus kit (MACHEREY-NAGEL). RNA-seq libraries were prepared using TruSeq RNA Library Prep Kit (Illumina) and sequenced with 75 bp single-end reads on Illumina NextSeq 500 (available in ArrayExpress under accession number E-MTAB-16368). Reads were aligned to the GRCh38 human genome using STAR alignment [25]. After filtering and generating count tables, differential expression was assessed using edgeR [26]. Gene set enrichment analysis (GSEA) was performed on a ranked gene list by *fgsea* package in R [27] working with KEGG and Gene Ontology gene sets. Statistical significance was determined by Benjamini-Hochberg FDR correction. Protein-Protein interaction networks were determined by STRING [28].

### Generation of mutated clones

A gRNA was designed per gene to target the ASD-related essential haploinsufficiency genes *PLAUR* and *PRKCA*. sgRNA sequences for *PLAUR* and *PRKCA* are GAAGATCACCAGCCTTACCG and GATGTGCAAGGAACACATGA, respectively. All sgRNAs were cloned into plasmid pSpCas9(BB)-2A-GFP (PX458, gift from Feng Zhang).

The cloned plasmids were transfected into h-pES10 haploid cells. Transfections were performed using polyethylenimine “Max” (PEI-Max, Polysciences) and OptiMEM (ThermoFisher Scientific) in a 1:2 DNA:PEI-Max and 2:25 PEI-Max:OptiMEM ratio, plated on MEF plates with hESCs growth medium and 10 μM ROCK inhibitor Y-27632.

48–72 h post-transfection, GFP-positive cells were isolated by FACS. Sorted cells were sparsely plated, and 7 days later, 24 colonies per gene were picked and expanded in 24-well MEF plates with hESC medium and ROCK inhibitor. Proliferating clones were transferred to 6-well MEF plates and picked up for sequencing. Two independent clones for each heterozygous and homozygous mutation for each gene were used for all downstream analyses.

### Sanger sequencing and potential off-targets analysis

Genomic DNA was extracted using the Blood & Cell Culture DNA Midi Kit (QIAGEN), followed by PCR amplification with gene-specific primers. *PLAUR:* forward - TGAAGATCCCCTCCTTGCAG, reverse - GATGAGGTTGAGCTGGGGAT. *PRKCA*: forward - TGCCATTGAGCTGACAGGTA, reverse - AAACGATCACTGGGGTTCCT. PCR products were resolved on 1.5% agarose gels at 120 V. Desired bands were excised and purified using HiYield Gel/PCR DNA Fragments Extraction Kit (RBC Bioscience). Sequencing was performed using the forward PCR primers on a QuantStudio 12 K Flex (Applied Biosystems). Mutations were documented in Figure S2A, and reads were visualized using SnapGene software (www.snapgene.com). Potential off-targets were identified using the CRISPOR web tool (www.crispor.gi.ucsc.edu). Notably, analysis of the most likely off-targets site in the 12 bp adjacent to the PAM sequence did not reveal any predicted off-targets sites with 0-3 mismatches. Hence, we used a more permissive potential off-targets threshold (all sgRNA length and up to 5 mismatches) for our analysis. Candidate off-targets located in coding regions and expressed in NPCs were selected for further validation. To assess these sites, IGV software was used to visualize RNA-seq data from the mutant clones and to quantify the fraction of reads matching the reference (WT) allele at the predicted off-target cleavage sites.

### Cell fusion

To establish the heterozygous clones, h-pES10 mutant clones for each gene were fused with h-pES12-dsRed haploid cells using the GenomeONE™ HVJ-E Cell Fusion Kit (Cosmo Bio USA), following the manufacturer’s instructions. Briefly, cells were trypsinized using trypsin-EDTA and manually counted. 2.5 × 10^5^ cells from each line were separately resuspended in 25 μL of 1:20 diluted GenomeONE fusion buffer, then mixed with 3 μL of HVJ-E in suspension. The mixture was incubated on ice for 5 min, followed by 15 min at 37 °C, then resuspended in mTeSR1 medium supplemented with 0.1% ROCK inhibitor Y-27632 and plated on Matrigel-coated 6-well culture dishes.

### Western blotting

The cells samples were lysed using 2XSDS + DTT buffer, incubated at 95 °C for 10 min and kept in −20 °C. Before use, lysates were reheated at 95 °C for 5-10 min followed by short spin down. Protein lysates were electrophoresed in 10% SDS polyacrylamide gels followed by wet transfer to nitrocellulose membranes that were then blocked with TBS-T buffer (0.15 M NaCl, 0.05 M Tris hydroxymethyl methylamine, and 0.1% Tween-20, pH 7.4-7.6) containing 5% nonfat milk (BD) for 30 min at room temperature. Membranes were then washed with a TBS-T buffer and incubated overnight at 4 °C with primary Antibodies (Abcam: ab218106 and Invitrogen: ma554043, for PLAUR and PRKCA accordingly), diluted according to manufacturer’s instructions. Membranes were then washed with TBS-T and incubated with secondary Ab (1:5000 in TBS-T with 4% nonfat milk) for 1 h at room temperature, followed by three washes with TBS-T, 10 min each. Then, chemiluminescence was visualized with a SuperPico ECL Master Mix reagent (Vazyme) using a Fusion FX gel imager. Results were quantified using the software programme Image Lab and normalized to the loading control (GAPDH).

### uPA–uPAR activation

To activate uPA–uPAR pathway in *PLAUR* heterozygous mutants, differentiating clones were supplemented with 50 ng/ml uPA (Sigma Aldrich, cat no: CC4000), added daily during the differentiation protocol.

### Establishment of essential haploinsufficiency genes

To calculate CRISPR scores for each gene and evaluate their impact on hESC growth, DNA-seq reads containing sgRNA sequences (read counts and depth for each replicate are listed in Table S1) were mapped by aligning sgRNA sequences to the reads using the Bowtie2 aligner [29], as previously described [21]. Following alignment, sgRNAs with zero reads in the control samples were excluded. A pseudocount of +1 was added to all read counts to allow for quantification of reduced sgRNAs. For increased statistical power, only genes targeted by more than three sgRNAs were included. Count tables were normalized relative to total reads per time point, and replicates were averaged. The CRISPR score for each gene was computed as the average log2 ratio of sgRNA abundance across the differentiated samples compared to the controls-undifferentiated samples cultured in parallel. Statistical significance was assessed using the two-sample Kolmogorov-Smirnov test (ks_2samp, SciPy stats module in Python), comparing each gene’s sgRNA distribution to the background sgRNA distribution. Genes with a CRISPR score < −1 and *p* value < 0.05 were considered significantly depleted. These were then filtered by comparison with the previously published neuroectodermal essential gene list [22] and only those with negative CRISPR scores in that dataset were retained. The count table was re-analyzed using the MAGeCK-test algorithm, using control sgRNAs for normalization [30].

### Cellular compartments

Protein localization data were downloaded from the Subcellular Localization Database (https://compartments.jensenlab.org/Downloads). Each gene was assigned to the compartment with the highest enrichment score (1–5). Genes associated with more than one compartment were excluded. Log2 ratios of extracellular matrix, plasma membrane and nucleus compartment representation were calculated for each of the conditions – HI NPCs, homozygous NPCs [22], HI ESCs [23] and homozygous ESCs [21] and normalized to the essential homozygous hESCs gene list and Fisher’s exact test was used for statistical testing.

### NDD and pathway analysis

To explore the role of NDD-associated genes in hESC growth, we used the OMIM database (https://www.omim.org/) to identify genes linked to NDDs, in combination with ASD-assosiated gene list obtained from SFARI Gene database (http.sfari.org/). NDDs and pathway enrichment analysis was performed on a ranked gene list using *fgsea* package in R [27], taking as input the composed NDD and ASD-assosiated gene lists along with KEGG and Gene Ontology gene sets. Statistical significance was determined by Benjamini-Hochberg FDR correction. Protein-Protein interaction networks were determined by STRING [28].

## Results

### Differentiation of the genome-wide haploinsufficiency mutant library into neural progenitor cells

**Fig. 1:**
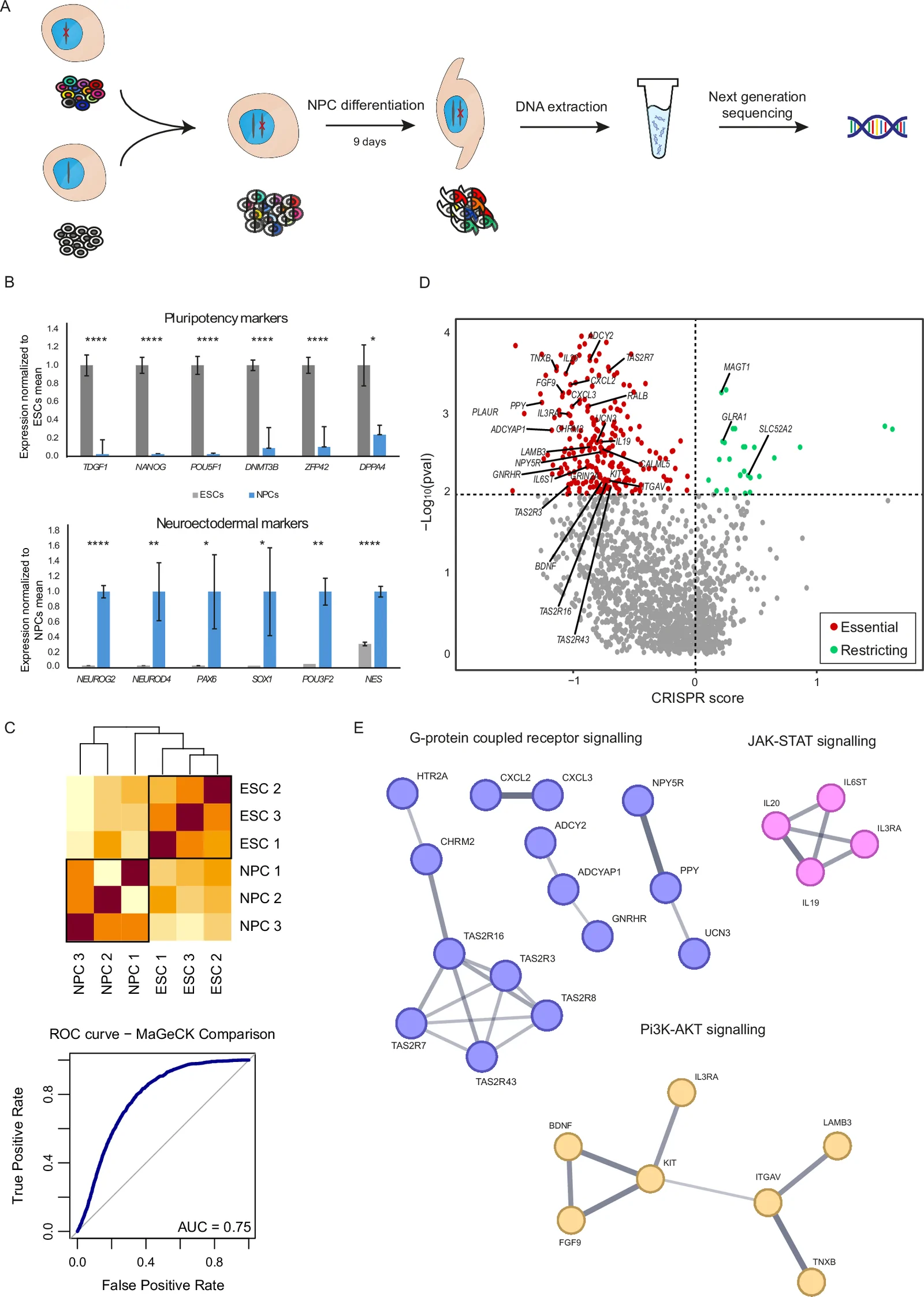
Differentiation of genome-wide haploinsufficiency CRISPR library into neural progenitor cells. **A** Scheme illustrating the establishment of the haploinsufficiency library and its differentiation into neural progenitor cells. **B** Expression of pluripotency and neuroectodermal markers in the undifferentiated (grey) and differentiated (blue) library cells (*n* = 3 for each category, independent replicates). FDR values were calculated by edgeR. **C** Heatmap deciphers correlation between the screens’ replicates as indicated by the darker colours, with a dendrogram demonstrating their Euclidian distance (top). ROC/AUC curve showing the concordance between the pipeline used in this study and MAGeCK hit calls (bottom). **D** Volcano plot representing the log_2_ *p* value and CRISPR score of all plasma membrane and extracellular matrix genes, marking essential genes (red), growth-restricting genes (green) and non-significant genes (grey). NDD-related essential genes are labelled. **E** Protein-protein interaction maps of pathways enriched with essential NDD-related genes. All genes were identified as depleted also in the MAGeCK analysis.

To identify genes essential for NPCs in a haploinsufficient manner, we utilized the genome-wide heterozygous loss-of-function mutant library (termed HI library) of human embryonic stem cells generated previously in our lab [23]. This library, composed of diploid human embryonic stem cells, was created by fusing two haploid cell lines—a WT haploid cell line [20], and a cell line containing a genome-wide loss-of-function library targeting all protein-coding genes [21]. The fusion resulted in a diploid genome-wide library, where each cell has one mutated and one normal allele for a single gene, covering most of the protein-coding genes in the human genome. We differentiated the HI library into neural progenitor cells using a 9-day protocol [22] (Fig. 1A and Methods), extracted DNA and RNA, and performed both DNA and RNA next-generation sequencing, achieving high library coverage with an average depth of more than 90 reads per sgRNA (Table S1), enabling robust detection of the haploinsufficiency mutations lead to depletion of the cells from the overall NPC population. Principal component analysis (PCA) revealed that the transcriptomes of the differentiated HI library samples were clearly distinct from those of the undifferentiated HI library, as they clustered separately (Figure S1A). Upon analysis of known pluripotency and neuroectoderm markers, the differentiated HI library samples were shown to downregulate main pluripotency markers such as *NANOG* and *POU5F1* and upregulate central neuroectoderm markers such as *NEUROG2, PAX6* and *SOX1*, compared to the undifferentiated HI library samples (Fig. 1B), indicating that the differentiation was successful.

Next, we aimed to assess gene essentiality during differentiation. We compared mutant cell abundance between the differentiated HI library samples and the undifferentiated HI library samples grown in parallel. The biological replicates showed high correlation across samples (Fig. 1C, top). For each gene, we calculated a CRISPR score (CS) [21] as the average log_2_(fold-change) in sgRNA abundance between NPCs and undifferentiated cells. Gene mutations with a negative effect on NPC growth and survival are expected to result in reduced sgRNA representation in differentiated cells, leading to a significantly negative CS for the gene and marking it as essential, while gene mutations leading to the expansion of the NPCs during differentiation will result in a significantly positive CS for the gene, marking it as restricting (Table S2). We also validated the concordance of our analysis with the MAGeCK pipeline, which showed similar biological effects for about 90% of all genes and 99% of the identified essential genes and a high AUC value, overall indicating a high similarity (Fig. 1C.

After composing the list of haploinsufficiency essential genes, we discovered that those genes code for proteins enriched in the extracellular matrix and plasma membrane, highlighting the importance of signalling pathways in NPC differentiation, especially in a dosage-dependent context (Figure S1B). In order to assess the effects of HI essential genes on signalling pathways in NPCs, we profiled the genes presented in the library located in the extracellular matrix and plasma membrane subcellular region (Fig. 1D). We discovered that our list of extracellular matrix and cell membrane essential genes (CS < 0 and pval < 0.05) was enriched with genes associated with various neurodevelopmental disease and further explored the pathways affected by these disease-related haploinsufficiency essential genes.

The top 3 pathways enriched with haploinsufficiency essential genes are G-protein coupled receptor (GPCR) signalling, Pi3K-AKT signalling and JAK-STAT signalling pathways, a result consistent with the MAGeCK analysis (Fig. 1E). These pathways have crucial roles in neural development [31–33], and all appear to be dosage sensitive. Interestingly, the JAK-STAT signalling pathway was previously shown to be enriched with haploinsufficiency essential genes also in the undifferentiated cell state [23], indicating this pathway as dosage-dependent in both undifferentiated and differentiated cell stages.

### Essentiality of neurodegenerative disease-related genes

**Fig. 2:**
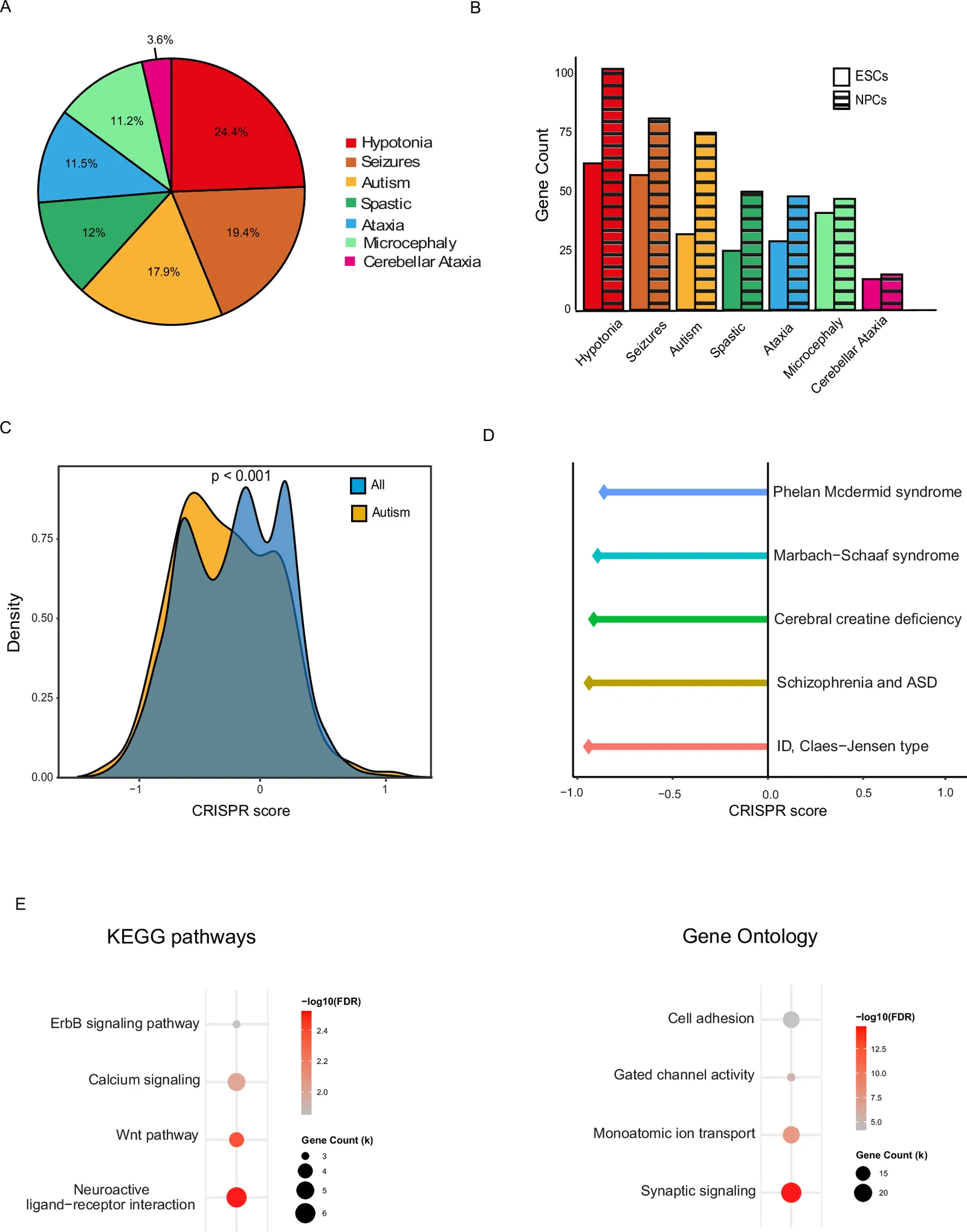
Analysis of haploinsufficiency essentiality in the context of neurodevelopmental diseases. **A** Pie chart illustrating the distribution of NDD-related essential haploinsufficiency genes for each type of disorder. **B** Gene counts of NDD-related genes in essential haploinsufficiency genes in and ESCs (empty) and NPCs (striped). **C** Density plot of CRISPR score distribution of ASD-related genes (yellow) and all genes (blue). *P*-value was calculated using *t*-test. **D** CRISPR score of genes related to specific ASD-associated diseases. **E** KEGG and Gene Ontology top results for downregulated terms enriched with ASD-related essential haploinsufficiency genes.

Next, we aimed to study the prevalence of neurodevelopmental disease-related genes among haploinsufficiency essential genes in order to identify disease-related genes with early haploinsufficiency effects. The haploinsufficiency essential gene list is significantly enriched with genes related to 7 neurodevelopmental conditions: hypotonia, seizures, autism, spasms, ataxia, microcephaly and cerebellar ataxia (Fig. 2A). These diseases showed higher representation of genes in haploinsufficiency essential gene list of NPCs than in ESCs (Fig. 2B).

ASD is a neurodevelopmental disease with complex genetic attributes [34]. Many of the ASD-related mutations occur de-novo in one of the parental gonads before fertilization, or post-fertilization in one out of two alleles at early developmental stages of the embryo, resulting in haploinsufficiency [35]. Thus, we decided to focus on ASD-related genes and their molecular phenotypes. When calculating the CRISPR score distribution of all ASD-related genes present in our HI NPC library, we revealed that the distribution is significantly lower than the overall distribution of all genes, meaning that they are relatively more essential (Fig. 2C). The negative CS tendency came across when focusing on specific genes associated with ASD-related syndromes, such as Marbach-Schaaf [36] and Phelan McDermid [37] syndromes (OMIM identifiers 619680 and 606232, respectively) (Fig. 2D).

Analysis of pathways and biological processes enriched with essential ASD-related haploinsufficiency genes (Fig. 2E) highlighted crucial neurodevelopmental molecular processes, such as cell adhesion and ion transport, along with important signalling pathways, such as Wnt and ErbB signalling, as significantly enriched with these genes, illuminating the molecular damages caused by ASD mutations occurring in early stages of neuroectoderm development.

### Transcriptional effects of heterozygous mutations of autism-related haploinsufficiency genes, *PLAUR* and *PRKCA*

**Fig. 3:**
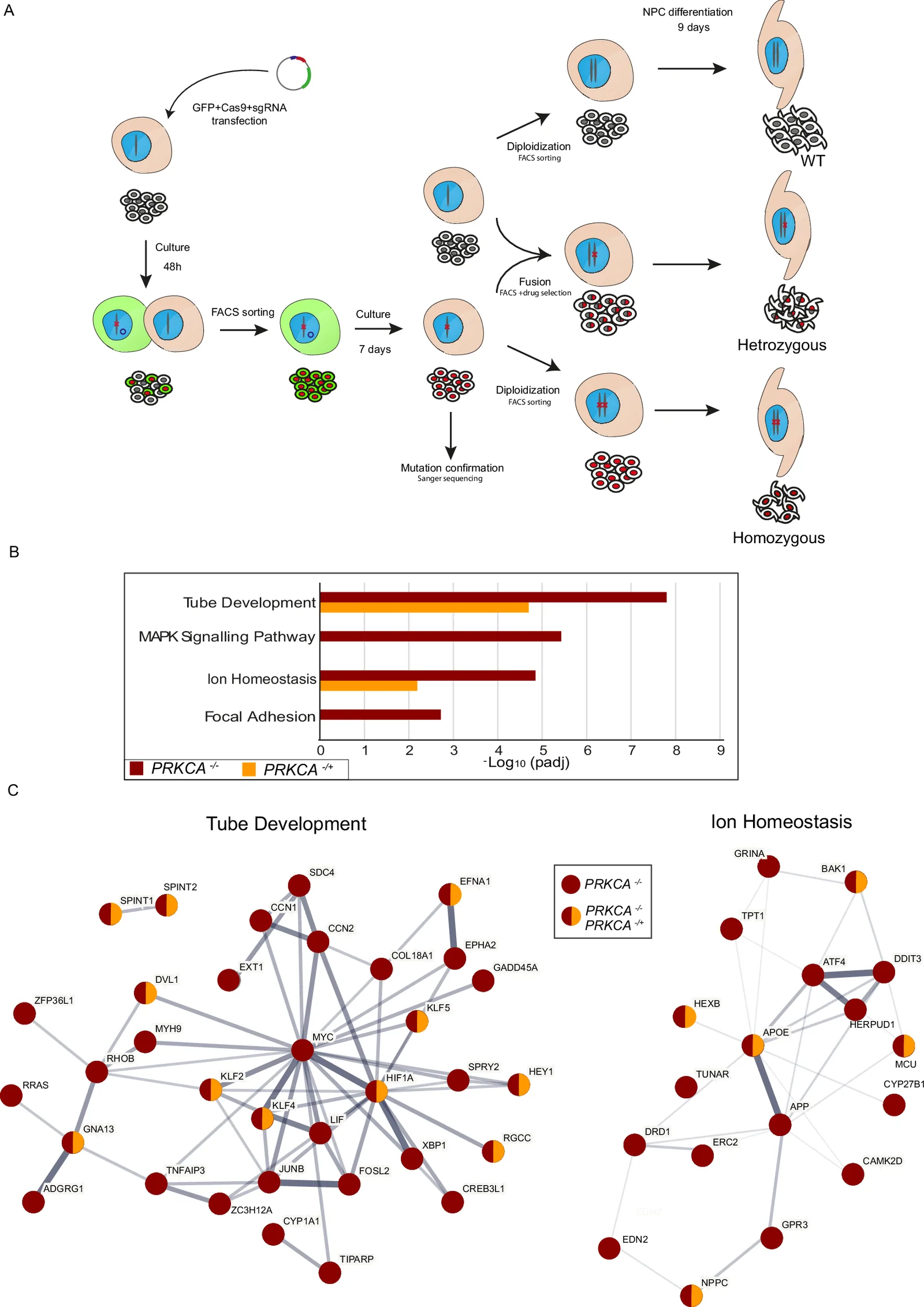
Establishment and analysis of *PRKCA* mutated clones. **A** Schematic illustration of the creation of homozygous and heterozygous clones. **B** Bar plots demonstrating the significance of downregulated biological processes of homozygous (dark red) and heterozygous (orange) mutated *PRKCA* clones. **C** Protein-protein interaction maps of genes downregulated in tube development and ion homeostasis. Red circles represent genes downregulated in homozygous clones. Red and orange circles represent genes downregulated in both homozygous and heterozygous clones.

To further investigate the importance of dosage in regulating cellular signalling pathways, we examined candidates for in-depth experimental analysis from the top essential ASD-related haploinsufficiency genes, including *PLAUR*, *MBOAT7*, *STX1A*, *SAE1*, *CCDC91* and *PRKCA*, ranked in that order (Table S3). We selected ASD-related candidate genes that are involved in cellular signalling pathways for further analyses. We focused on two ASD-related genes with low and significant CRISPR scores, which were also depleted in the MAGeCK analysis. The first gene was *PRKCA -* coding for protein kinase C alpha protein, which participates in many signalling pathways. The other gene was *PLAUR*, coding for urokinase plasminogen activator receptor (uPAR) protein, and an upstream regulator of several signalling pathways. We used CRISPR-Cas9 to create heterozygous and homozygous clones in hESCs and performed off-target analysis to ensure our perturbation specificity and correct interpretation of the mutant phenotypes (Fig. 3A and Figure S2A). Together with the genotypic validation, we additionally confirmed the dosage-dependent haploinsufficient phenotype at the protein level by western blot (Figure S3). Next, we characterized the transcriptomic shifts in each of the conditions following NPC differentiation and RNA-seq analysis. Importantly, analysis of differentially expressed genes in these samples revealed high reproducibility between replicates across independent clones (Figure S2B). While MAPK signalling pathway and focal adhesion were downregulated in *PRKCA*^*−/−*^ homozygous mutants only*, PRKCA*^*−/+*^ heterozygous mutants downregulated both tube development and ion homeostasis, both critical for proper neural development (Fig. 3B). Protein-protein interaction networks of these pathways demonstrated how certain genes’ expression (for example, *MYC*) were downregulated only in the homozygous mutants, while others (for example, the *KLF* homologs) were affected in both the homozygous and the heterozygous mutants in a dosage-sensitive manner (Fig. 3C).

**Fig. 4:**
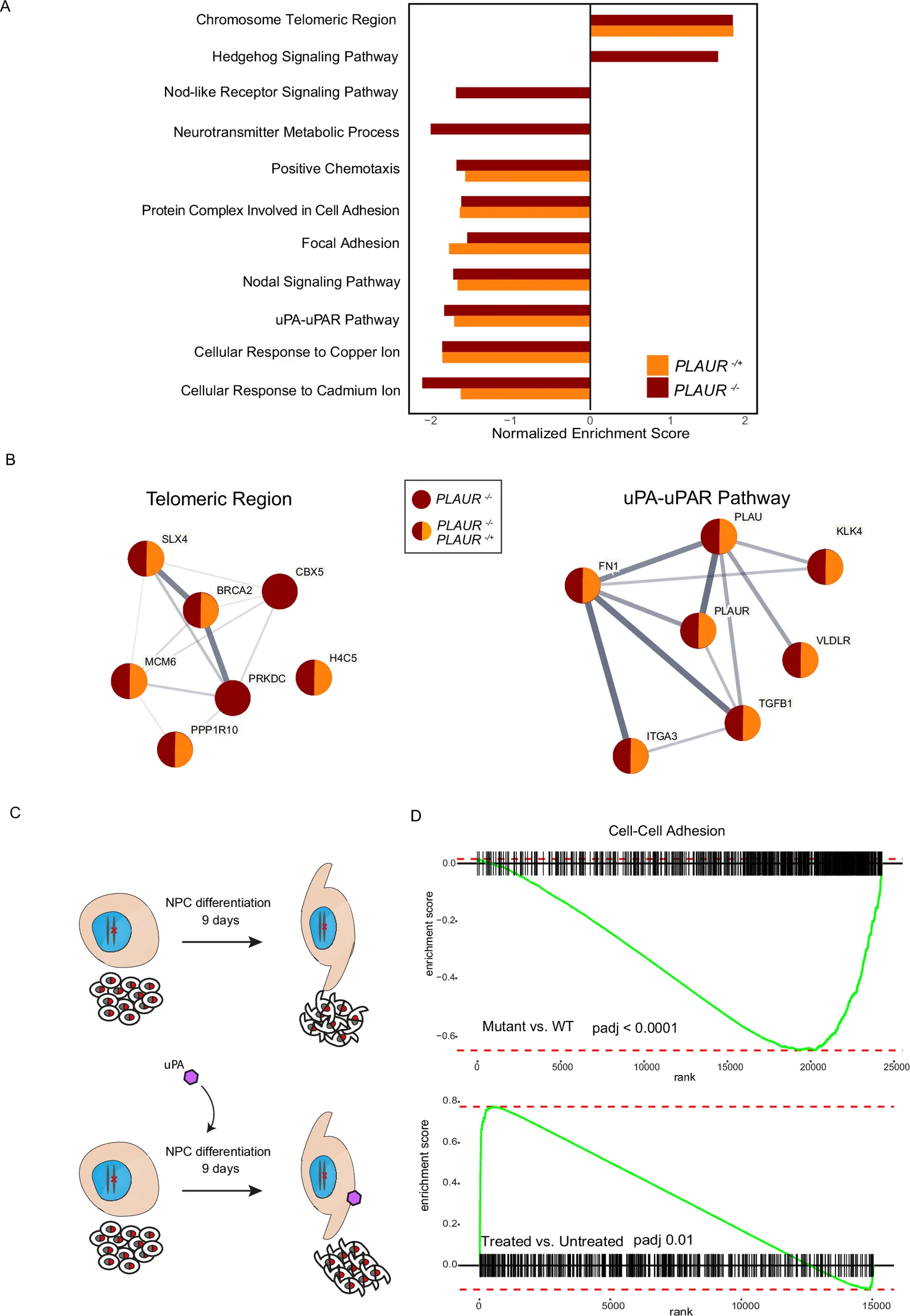
Analysis of the transcriptome and chemical rescue in *PLAUR* mutated clones. **A** Bar plots demonstrating the significance of downregulated biological processes of homozygous (dark red) and heterozygous (orange) mutate *PLAUR* clones. **B**. Protein-protein interaction maps of genes downregulated in uPA–uPAR pathway and upregulated in telomeric region. Red circles represent genes downregulated in homozygous clones. Red and orange circles represent genes downregulated in both homozygous and heterozygous clones. **C** Schematic illustration of chemical rescue experiment performed in heterozygous *PLAUR* clones. **D** GSEA enrichment plot of the cell–cell adhesion term in untreated (top) and treated (bottom) heterozygous *PLAUR* clones (*n* = 3 for each category, independent replicates).

*PLAUR*^*−/+*^ heterozygote mutants significantly altered several crucial processes, like downregulation of cellular response to various ions, NODAL signalling and cell adhesion, alongside upregulation of genes associated with telomeric regions. Hedgehog signalling pathway, NOD-like signalling and neurotransmitter metabolic processes were modified in homozygote *PLAUR*^−*\*^^−^ only (Fig. 4A). Protein-Protein interaction network revealed that all genes involved in the uPA–uPAR pathway were altered in both the homozygous and heterozygous mutants, while two genes associated with telomeric regions were only altered in homozygous mutants (Fig. 4B).

Based on the many essential pathways and molecular processes affected by *PLAUR* haploinsufficiency, we decided to further focus on the heterozygous mutants and attempt correction of the impaired molecular phenotype by activation of the uPA–uPAR signalling interaction. We have differentiated the *PLAUR*^-\+^ clones and externally added uPA, the protein binding to uPAR, to the medium daily, while parallelly differentiating the *PLAUR*^-\+^ clones without additional supplements (Fig. 4C). Differential expression analysis revealed that the major damaged phenotype, cell adhesion, had significantly improved in the NPCs that underwent the rescue treatment, along with upregulation of ion homeostasis, uPA–uPAR pathway and JAK-STAT signalling and downregulation of smoothened signalling pathway (Fig. 4D and Figure S4). Additional functional validation in the proteomic level could be valuable to further investigate the transcriptional haploinsufficiency phenotype we detected.

## Discussion

Our study thoroughly investigated the genetic and molecular effects of haploinsufficiency at early stages of neuronal development. By taking advantage of the exceptional genome-wide haploinsufficiency library, we have previously constructed in hESCs and differentiating it to neural progenitor cells, we are able to determine which genes, when heterozygously mutated, exhibit survival and differentiation disadvantage in a haploinsufficient manner.

Signalling pathways play a crucial role in the neuroectoderm differentiation process [22] as the differentiating cells depend on external signals for fate specification. Downregulation or disruption of these pathways may lead to impaired differentiation and survival of these cells. Extracellular matrix and plasma membrane are the compartments responsible for the initial communication of the cell with other cells and the outer environment and were shown to be especially enriched with haploinsufficiency essential genes in both hESC and NPCs, marking them as dosage-sensitive compartments in both undifferentiated and differentiated cell states.

Many mutations in genes associated with NDDs are haploinsufficient and show dosage sensitivity [9], prompting us to investigate the role of NDD-related genes in NPCs, one of the early stages of neural development. We discovered numerous genes that are involved in signal transduction and are essential for NPCs in a haploinsufficient manner. Further analysis revealed three important signalling pathways enriched with haploinsufficiency essential genes: GPCR signalling, Pi3K-AKT signalling and JAK-STAT signalling. GPCR signalling is crucial for proper neural development, as it is the largest transmembrane protein group responsible for many of the communication and signal transduction events of the developing neurons with the environment and plays a major part in both neurodegenerative [31] and neurodevelopmental [38] conditions, including motor impairments, epilepsy and others [38]. JAK-STAT is a central pathway in neural progenitor cells and is involved in proliferation, differentiation and even neurogenesis after injuries [33]. Pi3K-AKT is regulating many cellular events, essential for neural development and survival and involved in numerous NDD conditions, such as ASD, developmental delay and others[32, 39, 40]. Interestingly, we previously identified the Pi3K-AKT pathway as dosage-sensitive at the pluripotent cell state [23], which emphasize the dosage dependency of Pi3K-AKT signalling across different cell states. Notably, the high concordance of our findings with the re-analyzed data using the MAGeCK algorithm further reinforced the robustness of these results. The enrichment of haploinsufficiency essential NDD-related diseases in those pathways and in the outer cell compartments indicate not only the crucial part they play in NDD condition development once dysregulated, but also their importance to normal NPC development and survival and the dosage-sensitive mechanism by which they operate.

Upon analysis of NDD-related genes enriched within the haploinsufficiency essential gene list, we revealed 7 conditions. ASD was one of the top significantly enriched diseases and is known to be caused by haploinsufficiency in various genes. This highlighted the need to further focus on ASD in the haploinsufficiency context. We discovered that ASD-related genes show significantly lower CRISPR score than the general gene population represented in our library, and several molecular pathways and processes - such as cell adhesion, ion transport, ErbB signalling and others, with important roles in neural development were enriched with those genes. Notably, several genes associated with specific ASD and ASD-related disorders showed a significantly negative CRISPR score, suggesting these syndromes might have early haploinsufficiency effects at the neuroectodermal stage. There are many other ASD-related genes that did not appear to be essential in the NPC haploinsufficiency library, even though several of them have known haploinsufficiency effects. We presume that it is because they affect later stages of neuronal differentiation in the developing embryo and are involved in other molecular processes that do not significantly impair NPCs’ survival and differentiation. Moreover, the notion that some dosage-sensitive phenotypes may manifest during later stages of neuronal maturation or only in a 3D context highlights the importance of further studies to fully capture the spectrum of dosage-dependent effects, beyond early monolayer neuronal differentiation.

Interestingly, the two genes we focused on are both related to ASD but show different transcriptional shifts following their haploinsufficiency, indicating they affect early neural development through different dosage-sensitive mechanisms. Protein kinase C alpha, coded by *PRKCA*, is involved in many major signal transduction pathways crucial for correct neurodevelopmental processes, such as MAPK signalling, ErbB signalling, WNT signalling and others. The protein, along with other PKC isoforms, governs ion channels and ion homeostasis via phosphorylation [41]. Upon creating the heterozygous and homozygous mutated clones, we discovered that while MAPK signalling and focal adhesion were only downregulated in the homozygous mutants and not affected in *PRKCA*^*-/+*^, tube development and ion homeostasis were significantly downregulated in both heterozygous and homozygous mutants, marking these processes as dosage-sensitive, although those evidence are limited to the transcriptomic effects and further validation at the protein levels are needed. Intriguingly, *PRKCA* has also been considered an oncogene linked to the formation of cancerous brain tumours [42], supporting the notion that precise dosage control of *PRKCA* is critical for normal cellular function, as both its upregulation and downregulation can disrupt key regulatory pathways.

Plasminogen urokinase activator receptor (uPAR), coded by *PLAUR*, binds the urokinase plasminogen protein (uPA) and coordinates ECM proteolysis, thereby activating many cellular signalling pathways [43]. In addition, uPAR protein binds vitronectin located on the ECM of neighbouring cells, thereby regulating signalling pathways and cell–cell adhesion process [44]. The *PLAUR* gene was shown to be involved in neural migration, neurosphere survival and forming neural networks [45], and *PLAUR* disruption is related to ASD and other neurological pathologies [46–48]. When comparing the transcriptomes of the homozygous and heterozygous mutated clones, we discovered that many of the pathways and molecular processes - like cell adhesion, response to ions, chemotaxis and others - were significantly altered in both, highlighting the vast involvement of *PLAUR* in various dosage-sensitive biological functions. We attempted to correct the transcriptional changes caused by *PLAUR* haploinsufficiency and supplemented the heterozygous clones with external uPA protein. We indeed observed corrections of some of the transcriptional changes (Fig. 4D and Supplementary Figure 3), the most dramatic one being the impaired cell adhesion, but the downregulation of neural-related terms emphasized the importance of dosage balance in the uPA–uPAR pathway, as deviation in either direction will cause a phenotype, and further calibration of the system is needed before targeting the uPA–uPAR pathways as therapeutic target in ASD. Notably, our analysis of haploinsufficiency is primarily based on transcriptomic data. Although we detected the haploinsufficiency dosage effect in the protein levels for *PRKCA* and *PLAUR*, additional proteomic and functional assays could be beneficial to further investigate the observed haploinsufficiency effects. In summary, our unique genome-wide haploinsufficiency screen in differentiated human neural progenitor cells reveals that early neural development is fine-tuned by gene dosage and is dependent on various dosage-sensitive pathways. By linking haploinsufficiency to NDD–associated genes and pathways, we provide mechanistic insight into how genetic and transcriptomic imbalances can compromise normal neural function and early development. The distinct transcriptional effects observed for *PRKCA* and *PLAUR* haploinsufficiency mutations illuminate the diversity of dosage-sensitive mechanisms operating in early neural development. This study lays the framework for future research regarding other dosage-dependent mechanisms in ASD and other NDDs, paving the way for potential therapeutic approaches to treat these conditions.

## Supplementary information

Supplementary Figures and Legends

Supplementary Table 1

Supplementary Table 2

Supplementary Table 3

Uncropped western blot

## Data Availability

The datasets generated during the current study are available in the ArrayExpress repository, [https://www.ebi.ac.uk/biostudies/arrayexpress/studies/E-MTAB-16368]. Requests for further data should be directed to the corresponding author.
