# Supplementary material for: Analysis of haploinsufficiency in human neural progenitor cells: insights into early molecular effects of autism-related genes
Source: Cell Death Dis. 2026 Jul 8;17(1):847. doi: 10.1038/s41419-026-09085-2 (PMC13631298; doi:10.1038/s41419-026-09085-2)
Supplement: Supplementary file 1 [file 41419_2026_9085_MOESM1_ESM.pdf]

A

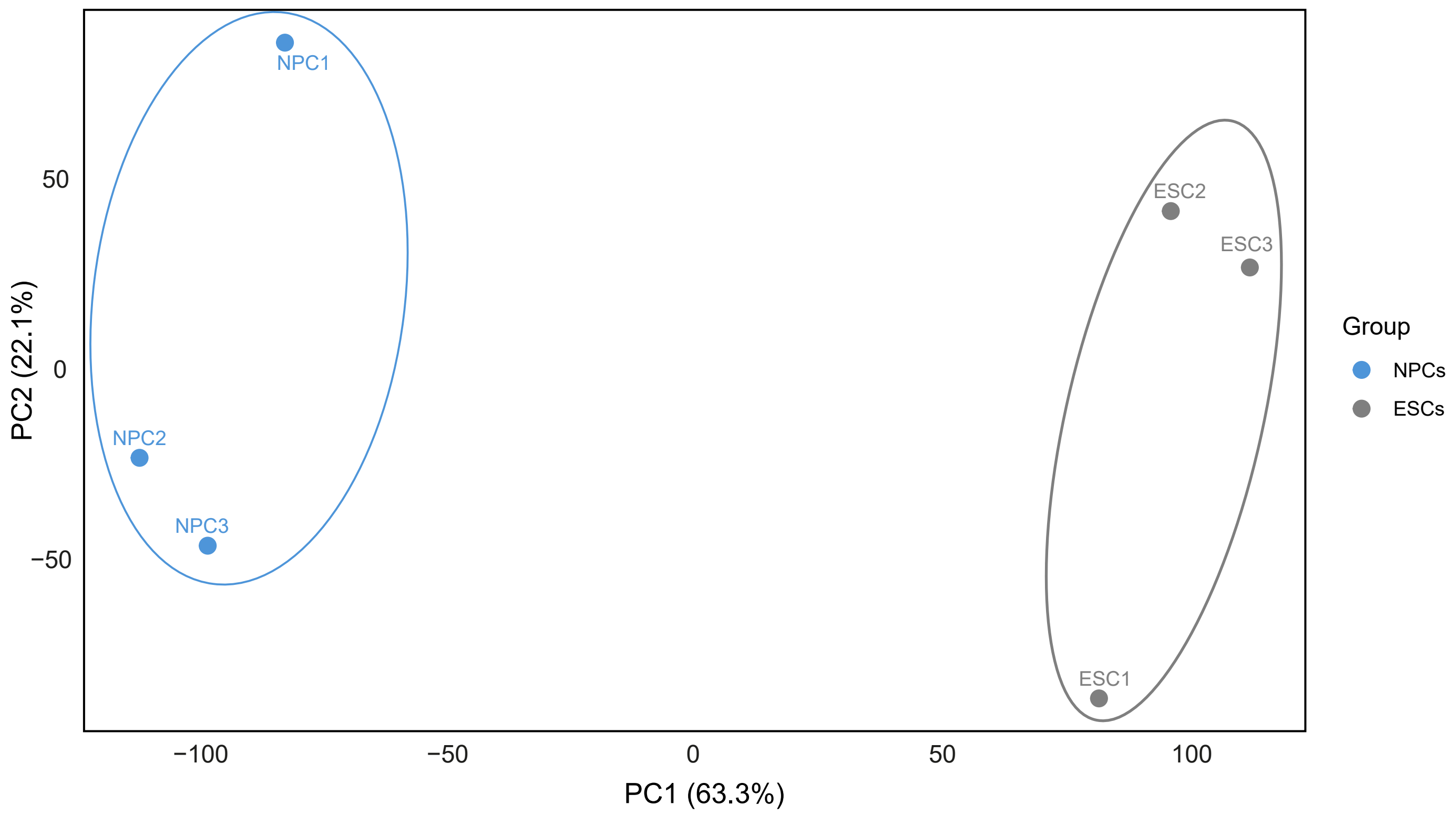

NPC1
NPC2
NPC3
ESC1
ESC2
ESC3
50
0
−50
PC2 (22.1%)
−100
−50
0
50
100
PC1 (63.3%)
Group
NPCs
ESCs

B

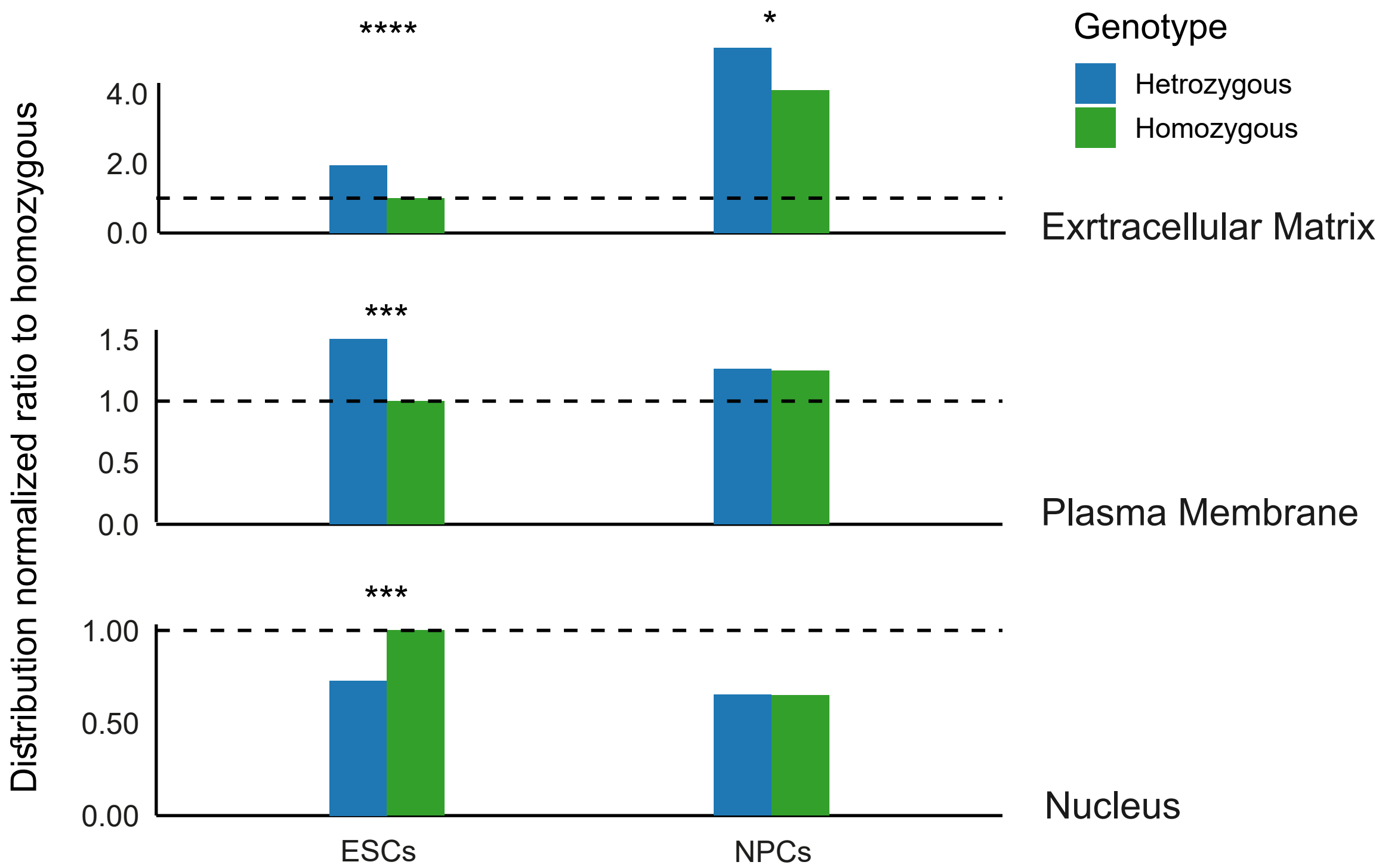

Distribution normalized ratio to homozygous
Genotype
Hetrozygous
Homozygous
****
*
4.0
2.0
0.0
Exrtracellular Matrix
***
1.5
1.0
0.5
0.0
Plasma Membrane
***
1.00
0.50
0.00
Nucleus
ESCs
NPCs

Figure S1-related to Figure 1 – Characterization of undifferentiated ESCs and their differentiated NPCs

A. Principal component analysis (PCA) plot of each haploinsufficiency library sample of undifferentiated (grey) and differentiated (blue) cells. B. Distribution of essential genes across extracellular, membrane and nucleus cellular compartments in homozygous (green) and heterozygous (blue) library CRISPR screens, in both ESCs (left) and NPCs (right). All values are normalized to homozygous ESCs.

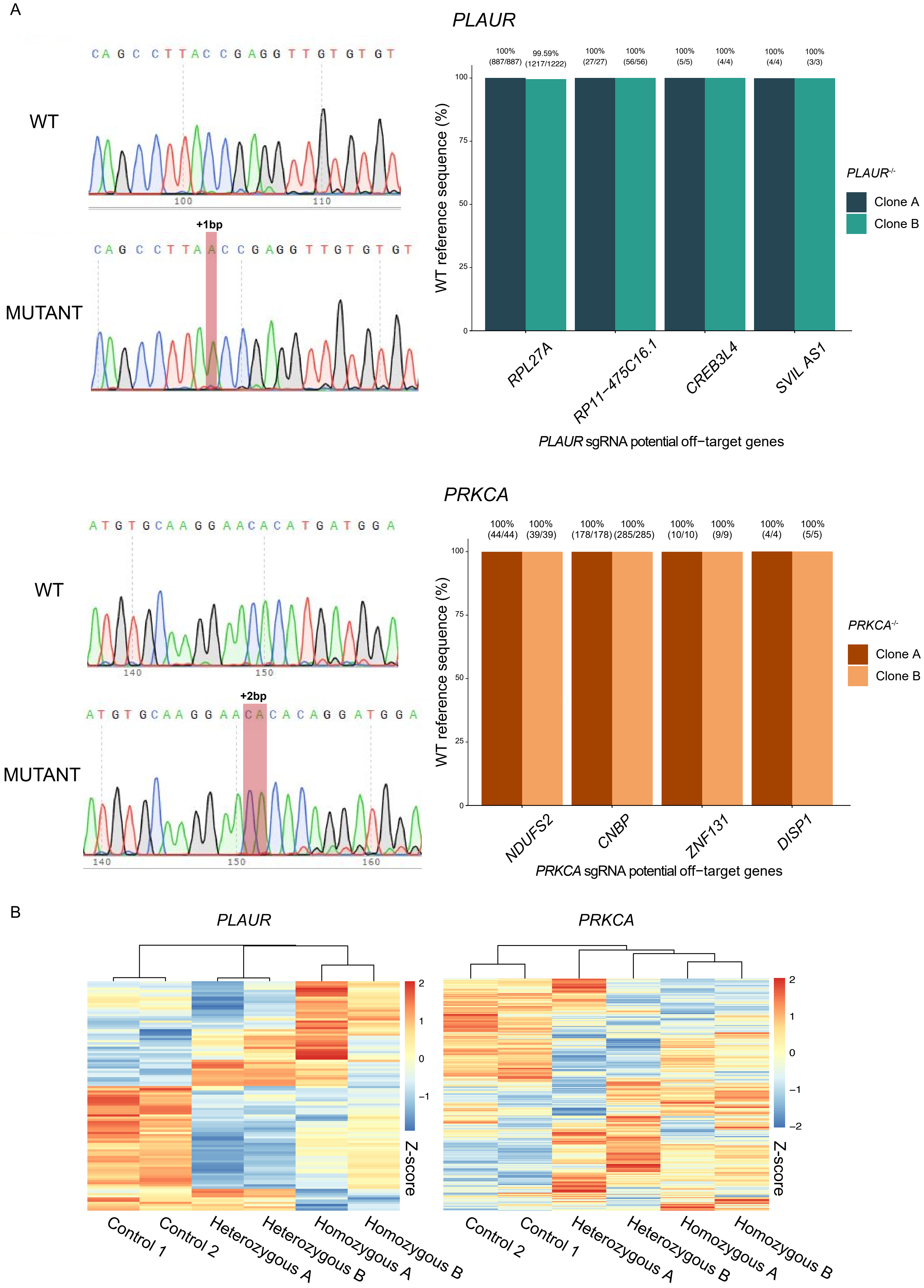

A
PLAUR
WT
MUTANT
+1bp
100
110
WT reference sequence (%)
100% (887/887)
99.59% (1217/1222)
100% (27/27)
100% (56/56)
100% (5/5)
100% (4/4)
100% (4/4)
100% (3/3)
PLAUR-/-
Clone A
Clone B
RPL27A
RP11−475C16.1
CREB3L4
SVIL AS1
PLAUR sgRNA potential off−target genes
PRKCA
+2bp
140
150
160
100% (44/44)
100% (39/39)
100% (178/178)
100% (285/285)
100% (10/10)
100% (9/9)
100% (4/4)
100% (5/5)
PRKCA-/-
NDUFS2
CNBP
ZNF131
DISP1
PRKCA sgRNA potential off−target genes
B
Z-score
Control 1
Control 2
Heterozygous A
Heterozygous B
Homozygous A
Homozygous B

Figure S2 – related to Figure 3 and 4 – Establishment and reproducibility of mutant clones

A. Sanger sequencing demonstrating the frameshift mutations (left) and potential off-targets analysis (right) of *PLAUR* (top) and *PRKCA* (bottom). B. Heatmap of the top differentially expressed genes in *PLAUR* (left) and *PRKCA* (right) mutants across replicates. Dendrograms on top demonstrating the clustering in Euclidean distance.

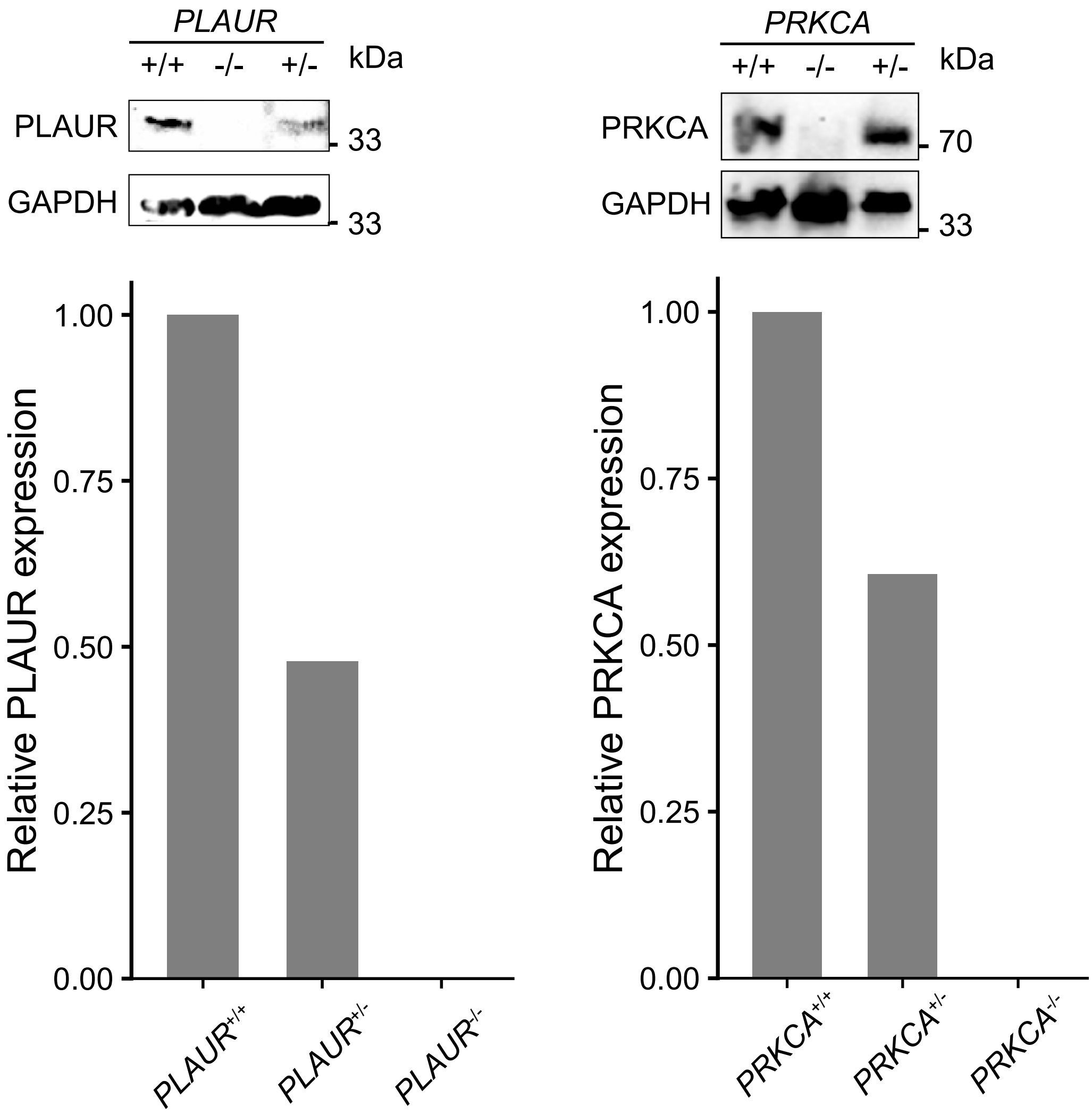
PLAUR
+/+ -/- +/-
kDa
PLAUR
GAPDH
33
33
PRKCA
+/+ -/- +/-
kDa
PRKCA
GAPDH
70
33
Relative PLAUR expression
1.00
0.75
0.50
0.25
0.00
PLAUR+/+
PLAUR+/-
PLAUR-/-
Relative PRKCA expression
1.00
0.75
0.50
0.25
0.00
PRKCA+/+
PRKCA+/-
PRKCA-/-

Figure S3 – realted to Figure 3 and 4 – Validation of haploinsufficiant phenotype at the protein level

Western blot analysis of homozygous, heterozygous and control *PLAUR* (left) and *PRKCA* (right) mutants, with histograms representation of the relative expression levels (bottom).

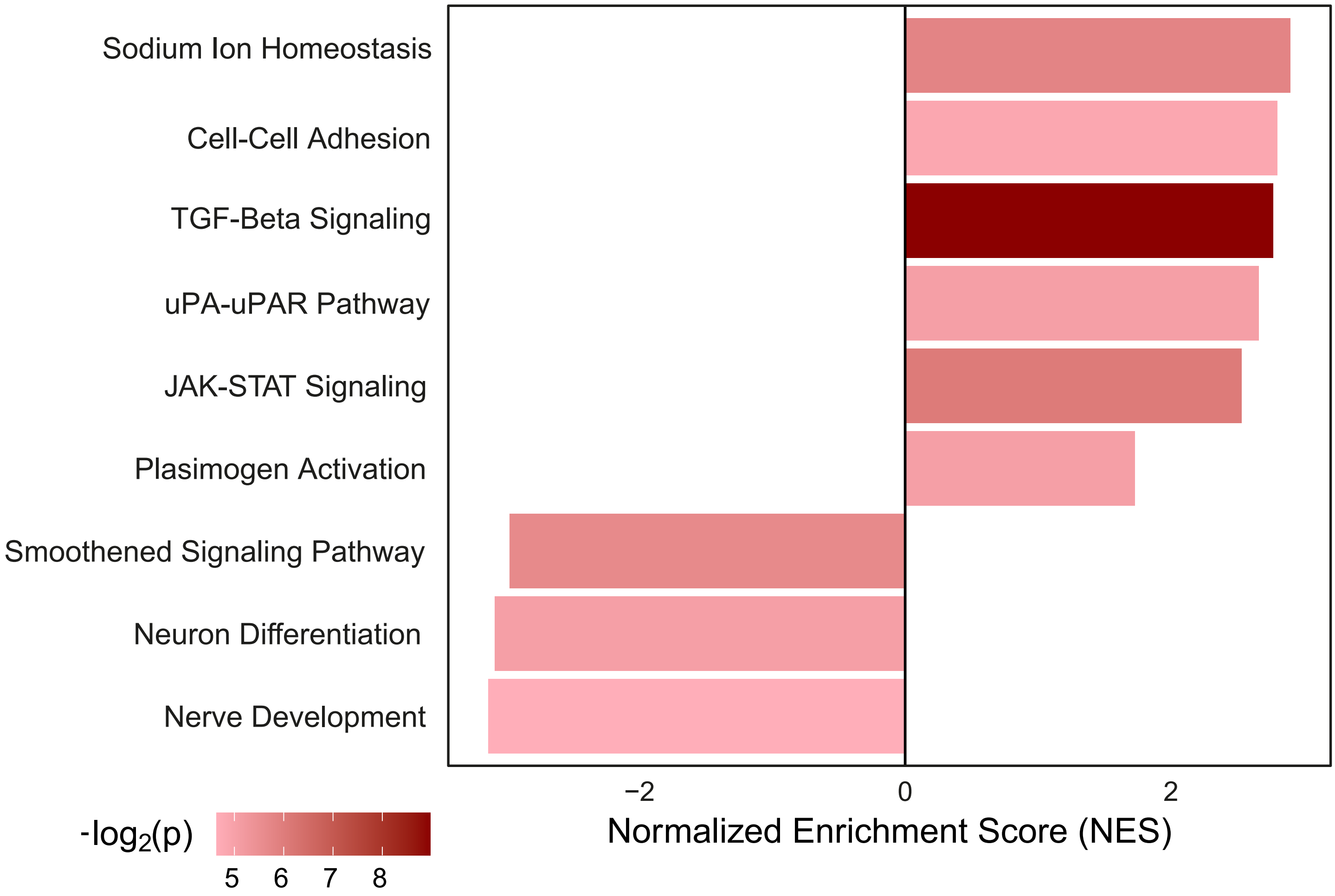

Sodium Ion Homeostasis
Cell-Cell Adhesion
TGF-Beta Signaling
uPA-uPAR Pathway
JAK-STAT Signaling
Plasimogen Activation
Smoothened Signaling Pathway
Neuron Differentiation
Nerve Development
−2
0
2
Normalized Enrichment Score (NES)
-log2(p)
5
6
7
8

Figure S4 – related to Figure 4 – Analysis of transcriptomic shift after uPA treatment

Bar plot demonstrating the normalized enrichment score of up- and down-regulated GO terms in the urokinase plasminogen activator (uPA)-treated heterozygous PLAUR clones, compared to untreated cells.

Supplementary Table 1

Single-guides library details, read counts coverage and depth statistics for the haploinsufficiency NPC library.

Supplementary Table 2

CRISPR scores, p-values and FDR for all genes represented in the haploinsufficiency NPC library.

Supplementary Table 3

List of top autism spectrum disorder essential haploinsufficient related genes.
