# Supplementary material for: Analysis of haploinsufficiency in human neural progenitor cells: insights into early molecular effects of autism-related genes
Source: Cell Death Dis. 2026 Jul 8;17(1):847. doi: 10.1038/s41419-026-09085-2 (PMC13631298; doi:10.1038/s41419-026-09085-2)

Original data - related to Figure S3

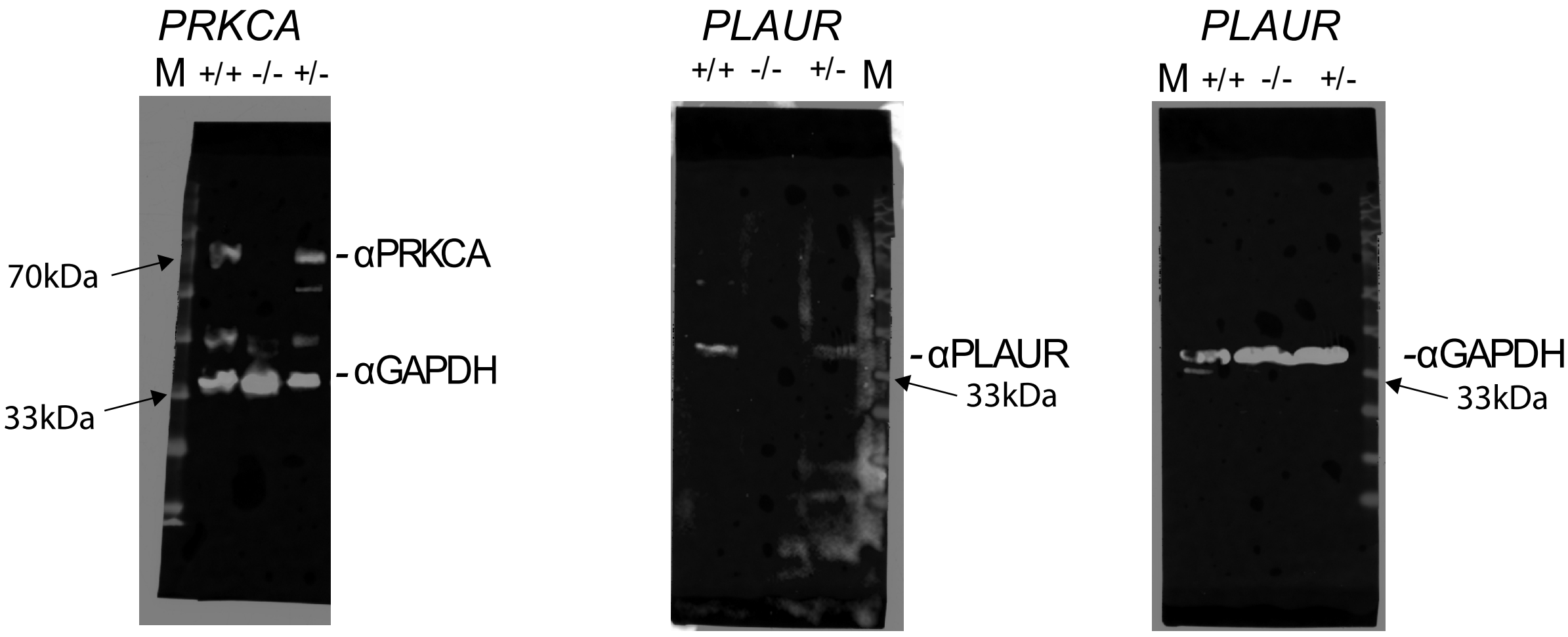

Supplement: Supplementary file 2 [file 41419_2026_9085_MOESM5_ESM.pdf]
